# Prevalence and unfavourable outcome of oral frailty in older adult: a systematic review and meta-analysis

**DOI:** 10.3389/fpubh.2024.1501793

**Published:** 2024-12-18

**Authors:** Sheng-Rui Zhu, Liu-Ying Wei, Kui Jia, Yuan-Xi Xie, Zheng-Ke-Ke Tan, Shu-Tian Mo, Wen-Zhen Tang

**Affiliations:** ^1^The First Affiliated Hospital of Guangxi Medical University, Guangxi Zhuang Autonomous Region, Nanning, China; ^2^School of Health Sciences, Universiti Sains Malaysia, Health Campus, Kubang Kerian, Kelantan, Malaysia; ^3^Nanning Fourth People’s Hospital, Guangxi Zhuang Autonomous Region, Nanning, China

**Keywords:** oral frailty, older adult, physical frailty, meta-analysis, outcome

## Abstract

**Background and objective:**

Oral frailty (OF) refers to a decline in oral function amongst older adult that often occurs alongside declines in cognitive and physical abilities. We conducted a study to determine the prevalence and unfavourable outcomes of OF in the older adult population to provide medical staff with valuable insights into the associated disease burden.

**Methods:**

From inception to March 2024, we systematically searched six key electronic databases: PubMed, Web of Science, Embase, Cochrane Library, Scopus, and CINAHL to identify potential studies that reported the prevalence or unfavourable outcomes of OF amongst older adult. Studies that did not have accessible data were excluded. Two researchers worked independently to retrieve the literature, collect data, and evaluate the quality of the included studies. Data analysis was conducted using R Project 4.1.1 and Review Manager 5.3 software.

**Results:**

We identified 28 studies that met the inclusion criteria, including 27,927 older adult. The pooled prevalence of OF amongst older adult was 32% (95% confidence interval (CI): 0.24, 0.41). Subgroup analyses indicated that the setting, sample, design of studies, and assessment instruments influence the prevalence of OF. In addition, OF was associated with a high risk of physical frailty (odds ratio (OR) = 1.67; 95% CI: 1.38, 2.02), malnutrition (OR = 2.27; 95% CI: 1.75, 2.96), low dietary variety (OR = 1.98, 95% CI: 1.15, 3.39), and social withdrawal (OR = 1.42; 95% CI: 1.18, 1.71).

**Conclusion:**

This systematic review and meta-analysis revealed that OF is prevalent amongst older adult. OF may affect the prognosis of older adult and thus necessitates comprehensive assessment and management as part of an integrated approach.

**Systematic review registration:**

https://www.crd.york.ac.uk/prospero/display_record.php?RecordID=537884.

## Introduction

The ageing population now poses a significant concern, marked by concurrent frailty and cognitive decline within this demographic group (1, 2). One critical priority is to effectively address the care challenges of older adults whilst concurrently mitigating the healthcare burden associated with an ageing population (3). Oral health is a global health priority, crucial for physical and mental wellbeing, and is a pivotal indicator of overall health during ageing (4). The prevalence of individual oral health issues tends to increase with age, and older adults commonly experience a multitude of coexisting oral health problems (5).

Recently, oral frailty (OF) has emerged as a significant concern in oral health and has been recognised as a geriatric syndrome requiring appropriate care, alongside well-known conditions, such as falls and physical decline (6). Factors related to OF in old age, including nutritional status and social aspects, are complex (5, 7). The concept of OF was introduced in Japan in 2013 to address issues related to oral function. Since then, measures to address OF have become a focus of Japan’s medical and welfare policies (8). According to the Japan Dental Association (2020), OF is defined as ‘a series of events and processes that contribute to changes in oral conditions (number of teeth, oral hygiene, oral functions, etc.) due to ageing (8). This is accompanied by a decrease in interest in oral health, reduced physical and mental capacity and an increase in oral frailty, leading to eating dysfunction. The outcome is a decline in both physical and mental function’ (8).

Significant variations exist in the prevalence of OF amongst older adult populations globally, ranging from 8.1 to 74% (9, 10, 53). This underscores the necessity for precise estimates of overall prevalence, which carry significant implications for the formulation of public health policies and resource allocation. Moreover, numerous studies have highlighted a robust association between OF and various adverse health outcomes in older adult. These include an increased risk of malnutrition, physical frailty, sarcopenia, long-term care needs, and premature mortality (3, 11, 52). However, different studies have various results. For example, two studies found that OF is related to low dietary variety (7, 12), but another finding indicated no significant correlation between the two (13). Furthermore, existing studies are constrained by small sample sizes, methodological variations in OF assessment, and geographic disparities. Further evidence is required to elucidate the relationship between OF and adverse health outcomes in older adults. This will provide a robust scientific foundation for developing interventions targeting the oral health needs of this demographic.

Therefore, this study aims to evaluate the incidence of OF and its associated unfavourable outcomes in older adult individuals through a systematic review and meta-analysis of existing research studies. The findings of this study will contribute to the development of targeted interventions and improve the overall health outcomes and quality of life in older adult populations.

## Methods

The study was preregistered with PROSPERO (registration no.: CRD42024537884) and conducted in compliance with the Preferred Reporting Items for Systematic Reviews and Meta-analyses guidelines.

### Search strategy

We systematically searched six critical electronic databases: PubMed, Web of Science, Embase, Cochrane Library, Scopus, and CINAHL, to find potential articles on the prevalence of OF or the unfavourable outcomes amongst older adult. We searched the database for articles published from inception until 31 March 2024. We also adopted a snowballing method to search the relevant literature. The primary search included terms related to OF and older adult (Supplementary Table S1).

Inclusion and exclusion criteria: inclusion criteria for this systematic review and meta-analysis were: (1) a cross-sectional or cohort study; (2) people aged ≥60 years; (3) reported the incidence of OF or unfavourable outcomes; and (4) published in English.

Exclusion criteria included (1) lacked statistics data; (2) publication in other languages; and (3) meetings, conferences, or reviews.

### Data extraction and quality assessment

First, the articles retrieved by the two researchers (SR Zhu and WZ Tang) from different electronic databases were combined in Endnote, and duplicate articles were removed. Second, the two researchers independently screened the titles and abstracts and reviewed the full text for eligibility. If the two researchers disagree, a third researcher (ST Mo) was called upon to make the ultimate conclusion. Finally, the two researchers independently extracted the following information: first author and publication year, study region, study design, sample size, age of participants, assessment tools, and the prevalence of OF.

In the cross-sectional study, the two researchers (SR Zhu and WZ Tang) used the research tool recommended by the Agency for Healthcare Research and Quality (AHRQ) (14). This tool consists of a total of 11 entries, each with ‘yes’, ‘no’, and ‘unclear’ as responses. Scores of 0–3, 4–7, and 8–11 denote low, medium, and high quality. The Newcastle–Ottawa Scale (NOS) recommended by the cohort study independently assessed the quality of each included study. It consists of 8 items, where the ‘comparability’ item is rated as 2 points, and all other items are rated as 1 point, with ≥7 points indicating higher literature quality (15).

### Statistical analysis

The pooled prevalence of OF in the older adult was summarised using a random effects model. The effect size was measured by prevalence, OR, and 95% CI. Heterogeneity amongst included studies was evaluated using the chi-square test and the I^2^ statistic, and the fixed effects model was used for meta-analysis when *p*-value was ≥0.100 and *I*^2^ was ≤50% (16).

To account for potential sources of heterogeneity in the prevalence of OF, we conducted subgroup analyses based on the characteristics of different study levels, such as study setting, sample size, and study design. In addition, for sensitivity analysis for each outcome, we used leave-one-out analysis or changed the merged model to evaluate the stability of the merge results.

Finally, funnel plots and Egger tests were used to evaluate the publication bias for the included papers. Data analysis was conducted using R Project 4.1.1 (New Zealand) and Review Manager 5.3 software (Copenhagen The Nordic Cochrane Centre, The Cochrane Collaboration). *p*-values were < 0.05, which indicated statistical significance.

## Results

### Search results

**Figure 1:**
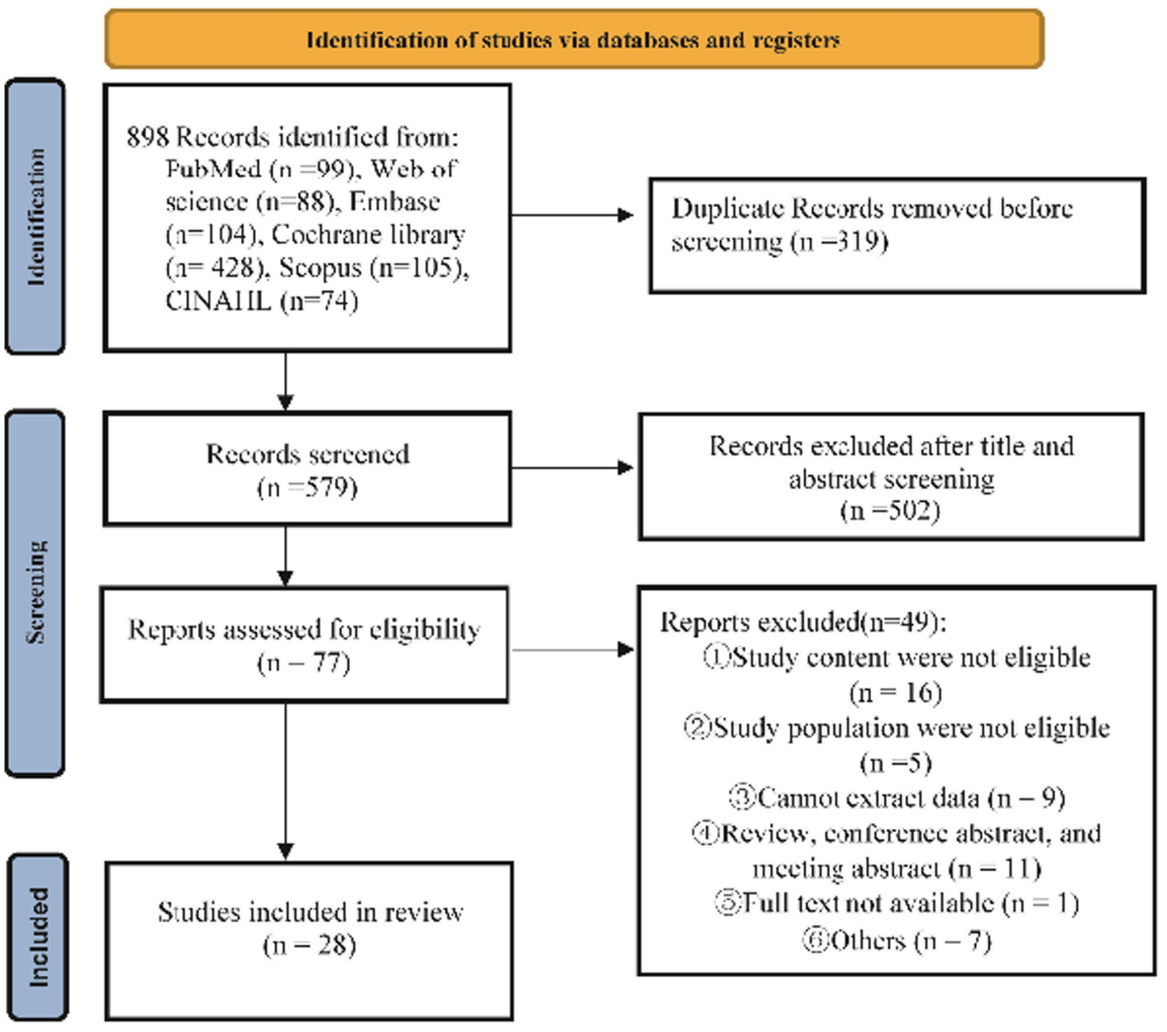
The literature screen flow chart.

We obtained 898 records from the 6 electronical databases. After eliminating 319 duplicates and carefully reviewing the titles and abstracts, we excluded 502 articles. After thoroughly reviewing the complete text of 77 articles, we were able to identify 28 studies that met our inclusion criteria (Figure 1).

### Included study characteristics

We included 28 papers in the study, involving 27,927 people. The included studies were published between 2016 and 2024, and most studies (*n* = 19) were conducted in Japan. Moreover, most studies (*n* = 23) were conducted in a community. In addition, most of the studies (*n* = 21) were cross-sectional, and a few were prospective cohorts (*n* = 7). The sample size of the participants in the included research ranged from 111 to 5,212.

The OF assessment tools included in the study included dental status, oral function, and subjective assessment. The OF standardised assessment tools included the OF Index-8 (OF-8), the OF 5-Item Checklist (OF-5), oral diadochokinesis (ODK), the Revised Oral Assessment Guide (ROAG), and Oral Health Assessment Test (OHAT). The prevalence of OF varied from 8.1 to 74%. Finally, the assessment of the literature quality resulted in scores ranging from 7 to 10, suggesting that the included literature was of excellent quality (Table 1).

**Table 1:** Basic characteristics of study.

First author, Year	Country	Settings	Study design	Sample size	Age	Tools	Prevalence %/score	Quality assessment score
Ayoob, 2024 (17)	India	Health centres	Cross-sectional	250	68 ± 6.02	Oral functions and dental status	74%	7
Baba, 2022 (9)	Japan	Community	Cross-sectional	210	74.2 ± 6.1	Dental status, oral functions and subjective assessments	8.10%	10
Chew, 2023 (18)	Singapore	Hospital	Cross-sectional	465	79.2 ± 8.3	ROAG	25%	9
Cruz-Moreira, 2023 (10)	Ecuador	Care homes	Cross-sectional	589	72 (66–82)	Oral function and subjective assessments	71%	10
Diaz-Toro, 2022 (31)	Chile	Community	Cross-sectional	1,186	≥60	Dental status and oral functions	–	8
Hasegawa, 2020 (19)	Japan	Community	Prospective	425	72.2 ± 5.6	Dental status and oral functions KCL.	49.60%	9
Hoshino, 2021 (7)	Japan	Community	Cross-sectional	481	75.9 ± 6.3	Dental status, oral functions and subjective assessments	21.20%	10
Ishii, 2022 (20)	Japan	Clinic	Cross-sectional	111	79.7 ± 3.8	OFI-8	53.20%	10
Iwasaki, 2020 (5)	Japan	Community	Cross-sectional	1,054	77 ± 4.8	Dental status, oral functions and subjective assessments	20.40%	10
Iwasaki, 2020 (11)	Japan	Community	Prospective	466	76.4 ± 4.1	Dental status, oral functions and subjective assessments	14.40%	9
Iwasaki, 2021 (21)	Japan	Community	Cross-sectional	1,082	77.1 ± 4.7	Dental status, oral functions and subjective assessments	21.00%	10
Iwasaki, 2024 (12)	Japan	Community	Cross-sectional	1,206	74.7 ± 5.5	OF-5	36.70%	10
Kamdem, 2017 (22)	Switzerland	Community	Cross-sectional	992	74.9 ± 1.39	Dental status and oral functions	14.80%	10
Kamide, 2023 (23)	Japan	Community	Cross-sectional	237	76.0 ± 5.7	OFI-8	54.90%	8
Kimble, 2023 (32)	United Kingdom	Community	Prospective	5,212	70–92	Dental status, oral functions and subjective assessments	–	9
Komatsu, 2021 (33)	Japan	Community	Cross-sectional	380	72.8 ± 5.6	Dental status, oral functions and subjective assessments	14%	9
Kugimiya, 2020 (24)	Japan	Community	Cross-sectional	679	76.3 ± 6.5	Dental status, oral functions and subjective assessments	22.50%	10
Kuo, 2022 (34)	Taiwan	Community	Cross-sectional	308	79.7 ± 7.2	Oral frailty checklist	4.87 ± 2.51	10
Nagatani, 2023 (25)	Japan	Community	Prospective	1,410	72.4 ± 5.2	Dental status, oral functions and subjective assessments	16.90%	9
Nishimoto, 2023 (26)	Japan	Community	Prospective	1,234	72.2 ± 5.1	Dental status, oral functions and subjective assessments	23.10%	9
Ohara, 2020 (13)	Japan	Community	Cross-sectional	722	79.1 ± 4.5	Dental status and oral functions	19.30%	10
Shimazaki, 2020 (27)	Japan	Community	Cross-sectional	978	65–85	Dental status, oral functions and subjective assessments	60%	10
Shwe, 2023 (35)	Australia	Hospital	Cross-sectional	115	80 ± 8.0	OHAT	4.3 ± 2.3	9
Tanaka, 2023 (28)	Japan	Community	Prospective	2031	73.1 ± 5.6	OF-5	39.30%	9
Tani, 2022 (36)	Japan	Community	Cross-sectional	381	72.6 ± 3.9	Dental status and oral functions and ODK	7.9 ± 1.2	9
Velázquez-Olmedo, 2021 (29)	Mexico	Community	Prospective	663	68.1 ± 6.1	Dental status and oral functions.	21.30%	9
Watanabe, 2016 (37)	Japan	Community	Cross-sectional	4,720	72.1 ± 5.6	Dental status and oral functions, and ODK	–	9
Yoshida, 2021 (30)	Japan	Community	Cross-sectional	340	75	Dental status, oral functions and subjective assessments	53.50%	9

ODK, oral diadochokinesis; ROAG, The Revised Oral Assessment Guide; KCL, Kihon checklist; OFI-8, The Oral Frailty Index-8; OF-5, Oral Frailty 5-Item Checklist; OHAT, Oral Health Assessment Test.

### Prevalence of OF in older adult

**Figure 2:**
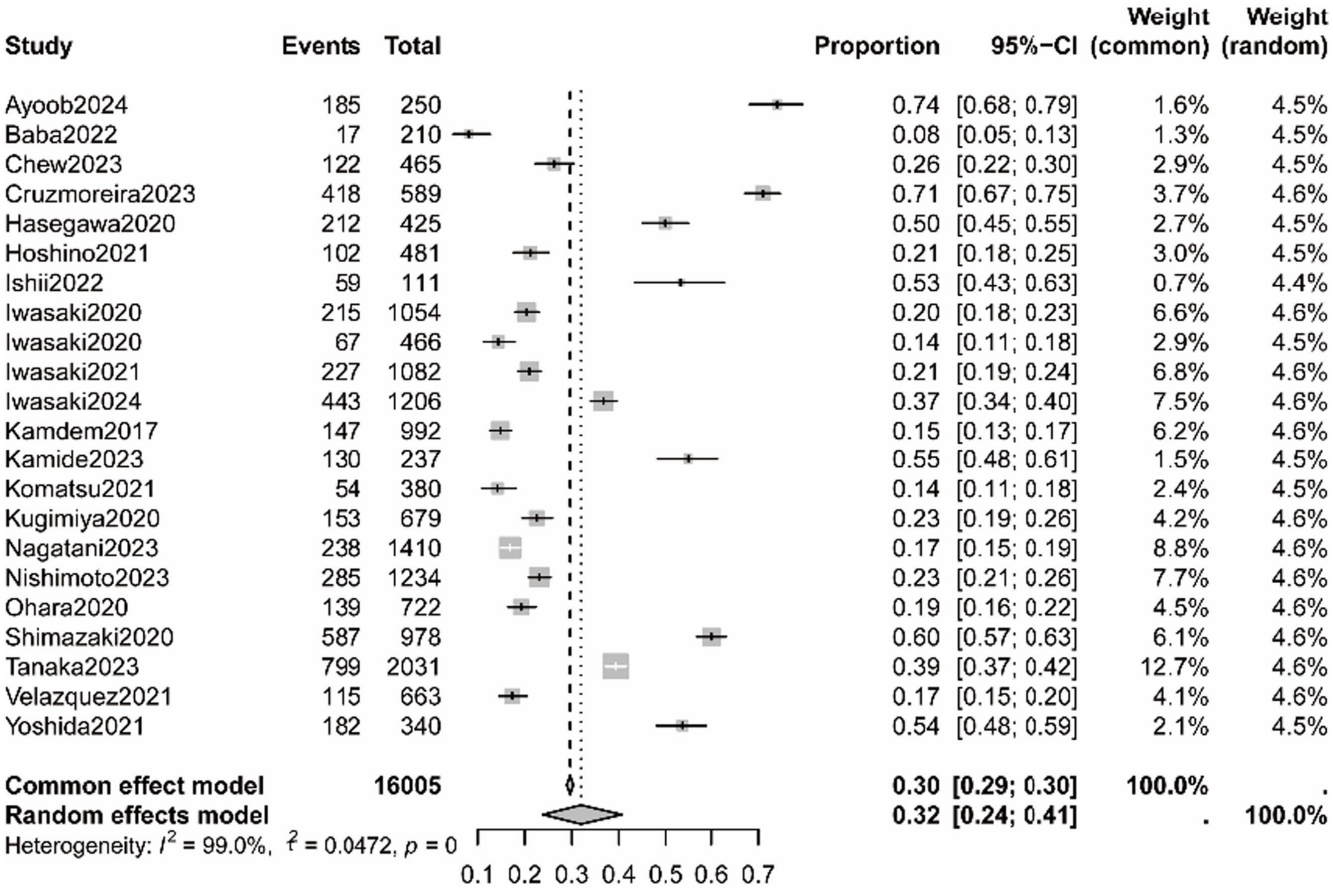
The pooled prevalence of oral frailty amongst older adult.

Based on 23 studies including 16,005 people (5, 7, 9–13, 17–30), the prevalence of OF was 32% (95% CI: 0.24, 0.41), with a high heterogeneity between 28 studies (*I*^2^ = 99%, *p* < 0.01; Figure 2). The results of sensitivity analysis showed no effect on the results after removing the literature one by one (Supplementary Figure S1).

The results of the Egger test are shown in Supplementary Figure S2. The results suggested no significant publication bias amongst the included studies (*t* = −0.50, *p* = 0.621). Moreover, Table 2 and Supplementary Figure S3 show the results of a subgroup analysis of OF prevalence. Significant interactions were found based on sample size (Supplementary Figure S3A), study setting (Supplementary Figure S3B), study design (Supplementary Figure S3C), and assessment tools (Supplementary Figure S3D). The OF was higher in non-community settings and studies with less than 1,000 samples. A high proportion of older adult was found to have OF in cross-sectional studies. Furthermore, studies using OFI-8 reported higher levels of oral frailty.

**Table 2:** Subgroup analysis for the prevalence of oral frailty.

Subgroup	Number of included studies	Prevalence (%)	95%CI	*I* ^2^	*p*-value
Study setting					
Non-community	4	56	0.34, 0.77	99	<0.01
Community	18	27	0.20, 0.35	99	<0.01
Study sample					
<1,000	16	34	0.23, 0.46	99	<0.01
≥1,000	6	26	0.19,0.34	99	<0.01
Study design					
Cross-sectional	16	34	0.24, 0.46	99	<0.01
Prospective	6	26	0.16,0.38	99	<0.01
Evaluation instruments					
OROAG	1	26	0.22, 0.30	–	–
OFI-8	2	54	0.49, 0.60	0	0.77
OF-5	2	38	0.36, 0.41	54	0.14
Other items	17	29	0.20, 0.40	99	<0.01

### Physical frailty associated with OF

**Figure 3:**
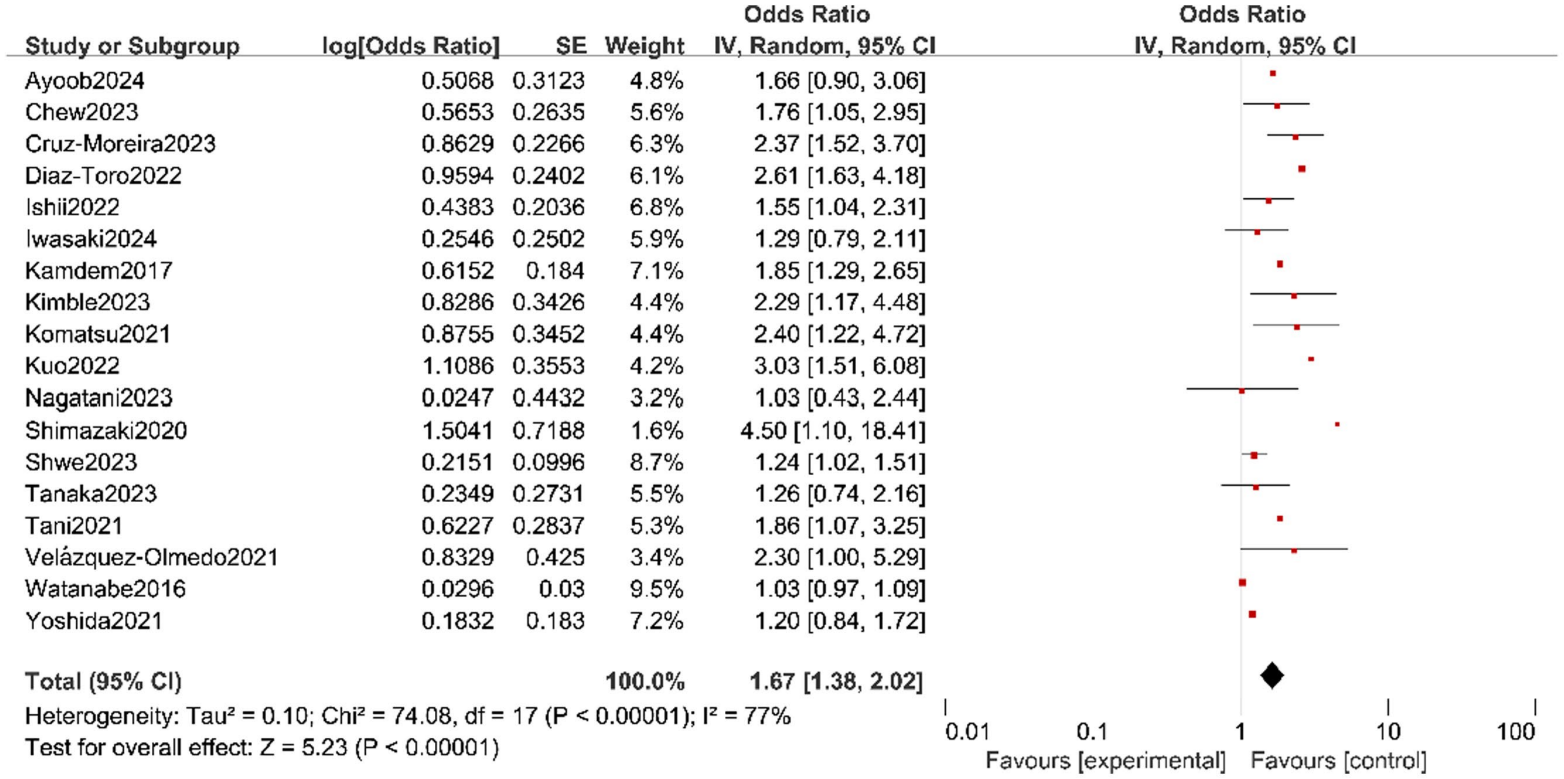
Forest plot of the pooled OR of physical frailty for oral frailty in older adult.

A total of 18 studies assessed the effect of OF on physical frailty in older adult (Figure 3) (10, 12, 17, 18, 20, 22, 25, 27–37). High heterogeneity was assessed amongst studies (*I*^2^ = 77%, *p* < 0.01), and the results of random effects models demonstrated that OF is positively associated with physical frailty (OR = 1.67, 95% CI: 1.38, 2.02). We further analysed the source of heterogeneity, and after excluding one study that was conducted in Japan (37), heterogeneity was reduced to *I*^2^ = 43%, which may be related to the variation in oral frailty instruments and participants’ characteristics. In addition, the funnel plot indicated no significant publication bias between the included studies (Supplementary Figure S4).

### Malnutrition associated with OF

**Figure 4:**
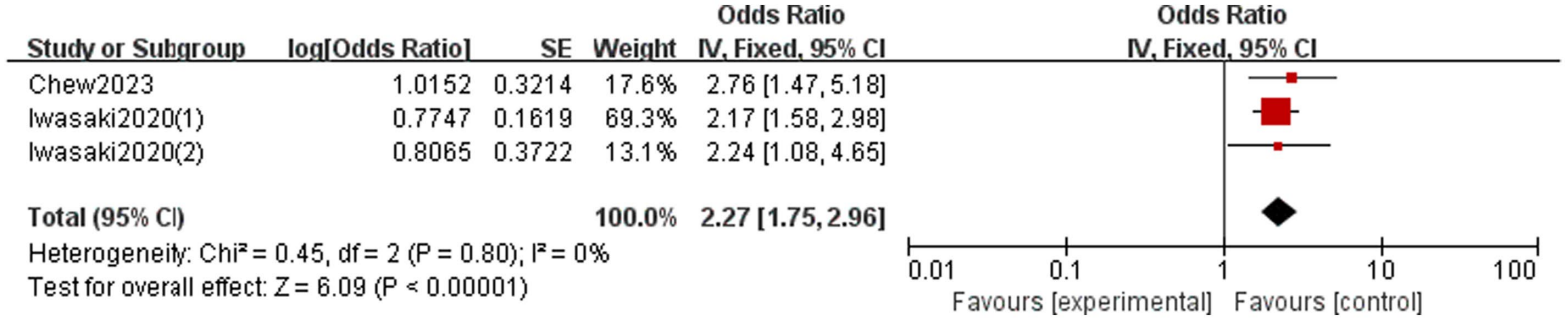
Forest plot of the pooled OR of malnutrition for oral frailty in the older adult.

Three studies reported the association between malnutrition and OF amongst the older adult (Figure 4) (5, 11, 18). No heterogeneity was assessed amongst studies (*I*^2^ = 0%, *p* = 0.80), and the results of fixed effects models demonstrated that OF can increase the incidence of malnutrition (OR = 2.27, 95% CI: 1.75, 2.96).

### Low dietary variety associated with OF

**Figure 5:**
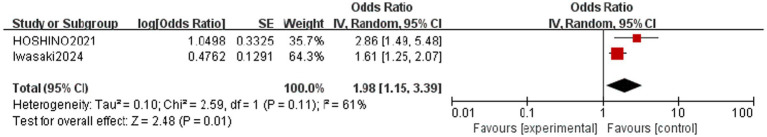
Forest plot of the pooled OR of low dietary variety for oral frailty in older adult.

Two studies assessed the association between low dietary variety and OF amongst the older adult (Figure 5) (7, 12). The random effects models indicated that OF is positively associated with low dietary variety (OR = 1.98, 95% CI: 1.15, 3.39).

### Social withdrawal associated with OF

**Figure 6:**
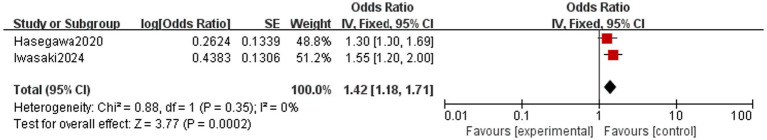
Forest plot of the pooled OR of social withdrawal for oral frailty in the older adult.

Two studies reported data on the relationship between social withdrawal and OF (Figure 6) (12, 19). No heterogeneity was assessed amongst studies (*I*^2^ = 0%, *p* = 0.35), and the results of fixed effects models indicated that OF can increase the incidence of social withdrawal (OR = 1.42, 95% CI: 1.18, 1.71).

### Low gait speed associated with OF

**Figure 7:**
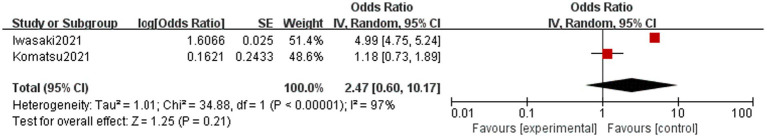
Forest plot of the pooled OR of low gait speed for oral frailty in older adult.

Two studies reported data on the association between low gait speed and OF (Figure 7) (21, 33). The results of fixed effects models showed no significant association between low gait speed and OF in the older adult (OR: 2.47, 95% CI: 0.60, 10.17).

### Sensitivity analysis

We conducted a sensitivity analysis by changing the effect model, and the results showed that the combined results of frailty, malnutrition, low dietary variety, and social withdrawal were consistent, indicating that the results were stable. However, the combined results of low gait speed were inconsistent (Table 3).

**Table 3:** Sensitivity analysis of oral frailty adverse outcome.

Outcome	Fixed-effect model			Random-effect model		
	*I* ^2^	OR (95%CI)	*p*-value	*I* ^2^	OR (95%CI)	*p*-value
Physical frailty	77	1.11(1.06, 1.16)	<0.01	77	1.54(1.33,1.79)	<0.01
Malnutrition	0	2.27(1.75, 2.96)	<0.01	0	2.27(1.75,2.96)	<0.01
Low dietary variety	61	1.74(1.37, 2.20)	<0.01	61	1.98(1.15,3.39)	0.01
Social withdraw	0	1.42(1.18, 1.71)	<0.01	0	1.42(1.18,1.71)	<0.01
Low gait speed	97	4.91(4.68, 5.16)	<0.01	97	2.47(0.60,10.17)	0.21

### Descriptive analysis

The following outcomes were reported in only one study, so descriptive analysis was used to report the effect of OF on them. OF was found to be associated with the following unfavourable outcomes: disability (OR = 1.40, 95%CI: 1.14, 1.72) functional decline (OR = 1.61, 95% CI: 1.01, 2.59) (18), oral candidiasis (OR = 1.72, 95% CI: 0.44, 6.70) (9), short stride length (OR = 0.23, 95% CI: −2.72, −1.05) (21), short step length (OR = 3.31, 95% CI: 0.53, 21.53) (21), wider step width (OR = 0.20, 95% CI: 0.12, 0.33) (21), longer double support duration (OR = 2.84, 95% CI: 1.70, 4.74) (21), higher stride time (OR = 2.29, 95% CI: 0.56, 9.41) (21), and fall risk (OR = 2.38, 95%CI: 1.11, 5.07) (23).

## Discussion

In terms of the literature, this meta-analysis and systematic review is the first to evaluate the overall prevalence of OF and its unfavourable outcomes in older adult. Overall, the findings point to a notable prevalence of OF in the older adult, which is associated with an increased risk of physical frailty, malnutrition, low dietary variety, and social withdrawal.

OF refers to the progressive age-related loss of oral function caused by a range of injuries that leads to the deterioration of daily oral function in older adults and is significantly associated with many of their adverse events (8). Geriatrics specialists have been emphasising on frailty in the field of healthcare recently (38). Our meta-analysis, comprising 28 research with a total of 27,927 older adults, revealed a pooled OF at 32% (95% CI: 0.24, 0.41), similar to a previous study (39). A recent systematic review demonstrated that the prevalence of OF was 24%, ranging from 20 to 28% (39). This demonstrates that OF is a prevalent condition amongst older adult and may be directly linked with the decline in oral function that often accompanies ageing (8).

The results of the subgroup analysis of our study also demonstrated that older adult in hospitals experience a higher incidence of OF than in communities, which may be related to the hospital environment and treatment factors. Hironaka et al. discovered that the incidence of OF increased with age amongst 682 older adults who lived in the community, and the age of the older adult with OF (77.1 ± 6.5) was significantly higher than that of the robust group (71.1 ± 5.7) (40). This conclusion that the prevalence of OF increases with age was also confirmed in another study (41). Therefore, by evaluating the prevalence of OF, our study can raise awareness amongst healthcare providers and enhance their understanding and assessment of this condition in the older adult population, which will contribute to improving the overall health of older adult and facilitate the development of healthcare interventions and related disciplines targeting OF.

Additionally, studies with larger samples reported lower levels of prevalence of oral frailty, compared with study samples fewer than 1,000. This may be due to variations in the assessment instruments used for oral frailty and the greater population diversity in larger samples. Finally, our meta-analysis indicated that studies with different designs have different levels of oral frailty. Cross-sectional studies can only capture data on oral frailty at a single point in time, whilst longitudinal studies may track the development or changes in oral vulnerability over time, potentially leading to different findings. Furthermore, a higher prevalence of oral frailty was observed in the study using the OFI-8 than other instruments, suggesting that this instrument may be more sensitive in identifying older adult with oral frailty.

In addition, the results of the sensitivity analysis showed that excluding each study one by one had no effect on the overall results, and enhanced the reliability of our conclusions.

In our study, OF is associated with a higher risk of physical frailty amongst older adult, similar to previous studies (3, 42). A systematic review by Dibello et al. demonstrated that physical frailty was associated with indicators, such as worsening oral health and chewing and swallowing disorders, and amongst them, the most commonly reported indicators are the number of teeth and chewing disorders (3). The connection between OF and physical frailty has multiple explanations. First, the first possible cause is an inflammatory pathway. A study confirmed a significant relationship between inflammation and frailty, that is, an inflammatory state amongst the older adult will reduce their muscle’s ability to synthesise protein and ultimately increase their risk of dysfunction (43). However, the evidence supporting the existence of inflammatory pathways is not strong, as a study indicated that no correlation exists between periodontal markers and frailty (44). Another possible reason is the effect of OF on nutrition and food intake. Previous research has demonstrated that malnutrition acts as a mediator in the relationship between oral health and the impairment of physical development (44, 45). Older adult individuals with OF frequently encounter issues such as diminished tooth count, challenges with chewing, and periodontal disease. These problems can result in eating difficulties, leading to malnutrition, and ultimately contributing to the development of physical frailty in the older adult (5). Furthermore, we found that OF may increase the risk of malnutrition and low dietary variety, which is consistent with the study of Hussein et al. (46). Their systematic review, which included a total of 33 studies, confirmed that older adults with poor daily oral hygiene, chewing problems, and partial/complete toothlessness are at higher risk of malnutrition (46). OF in the older adult may be due to reduced number of teeth, chewing and swallowing difficulties, and oral pain, leading to reduced food intake and variety, thereby resulting in malnutrition. Edentulous patients have been reported to consume less fibre and carotene and more cholesterol and saturated fat than patients with 25 or more teeth (47). Of all the pathophysiological mechanisms leading to malnutrition, oral hygiene may play a key role (48). In addition, dry mouth disease and pathological denture-related diseases are common in the older adult (49), which may cause their appetite to decrease, carry on the interest that affects eating, and eventually lead to the occurrence of malnutrition. However, a clear and direct association between inadequate OF and malnutrition remains lacking, specifically in terms of the underlying pathogenic mechanisms. Therefore, these findings emphasise the significance of OF and further illustrate that precise and sufficient oral screening and assessment are crucial in the comprehensive management of older adult.

Social withdrawal is also one of the unfavourable consequences of OF amongst the older adult, that is, older adult with OF are likely to report social withdrawal. A previous study has indicated that the older adult have a decrease in their motivation and capacity to leave their homes due to a combination of physiological and psychological variables (50). These issues include a deterioration in physical function, which is often influenced by concerns about their looks and OF. In addition, some studies have indicated that the opportunity to go out often involves eating and beverages, which might pose challenges for the older adult with OF, perhaps leading to eating and swallowing difficulties (19, 51). Given their limited dietary options, individuals must take breaks whilst eating, which can result in psychological strain and subsequently have adverse effects on their social life.

Overall, our study identifies some significant knowledge deficiencies about OF in older adult individuals and has significant practical implications for the field of medicine and public health. First, OF should be considered a significant component of baseline evaluation in older adults in clinical settings, and the applicability value of various assessment tools for OF in older adults can be further compared. Second, healthcare professionals specialising in clinical care can evaluate the level of OF in older adult individuals and create personalised strategies to enhance primary healthcare and offer comprehensive treatment for OF in older adults. For instance, healthcare professionals could offer education and training on oral health care for the older adult, including guidance on nutrition, effective oral self-care practises, oral infection management strategies, and exercises designed to enhance oral function; provide the training to family members or caregivers on how to assist with oral care. In addition, future primary research should expand the geographical diversity of study samples of the older adult to enhance the generalizability of the results.

Furthermore, our study has significant implications for future research. First, future research should focus on the development and validation of standardised assessment tools for oral frailty to ensure consistency, reliability, and comparability across studies. Second, we suggest that future studies should conduct more international cohort studies, to ensure that results are applicable across regions and diverse populations. In addition, future studies could investigate more outcomes to examine the impact of oral frailty on health, including cognitive function and comorbidities.

Our study has significant strengths. First, we performed a quantitative meta-analysis of 28 studies from 10 countries, encompassing a total of 27,927 older adult people. This meta-analysis is the most extensive systematic synthesis ever conducted in this particular field. Second, we used AHRQ and NOS assessment instruments to appraise the quality of the literature included, and the findings indicated that the included literature demonstrated a high level of quality. Furthermore, we analysed prevalence based on the study setting, sample, and type to determine any variation in estimates.

Nevertheless, our study also has several limitations. First, the included studies in the meta-analysis utilised different assessment instruments for OF, and some of the research used subjective instruments, which could be one of the major causes of heterogeneity. Furthermore, we limited our inclusion to studies published in English, which might have led to an incomplete inclusion. Moreover, the majority of the research included in our analysis was conducted in Japan (*n* = 19), indicating that our findings may have limited applicability and should be interpreted carefully.

## Conclusion

The purpose of this systematic review was to determine the prevalence of OF and its unfavourable outcome amongst the older adult. The results indicated a high prevalence of OF amongst older adult. OF is associated with a poor prognosis for the older adult. Therefore, healthcare professionals should prioritise the evaluation and management of OF in older adults. They should also create effective strategies to improve the overall health and lifespan of older adult.

## Data Availability

The original contributions presented in the study are included in the article/Supplementary material, further inquiries can be directed to the corresponding authors.

## References

[1] Feng L Nyunt MS Gao Q Feng L Lee TS Tsoi T . Physical frailty, cognitive impairment, and the risk of neurocognitive disorder in the Singapore longitudinal ageing studies. J Gerontol A Biol Sci Med Sci. (2017) 72:369–75. doi: 10.1093/gerona/glw050, PMID: 27013397

[2] Waite SJ Maitland S Thomas A Yarnall AJ. Sarcopenia and frailty in individuals with dementia: a systematic review. Arch Gerontol Geriatr. (2021) 92:104268. doi: 10.1016/j.archger.2020.104268, PMID: 33011431

[3] Dibello V Lobbezoo F Lozupone M Sardone R Ballini A Berardino G . Oral frailty indicators to target major adverse health-related outcomes in older age: a systematic review. GeroScience. (2023) 45:663–706. doi: 10.1007/s11357-022-00663-8, PMID: 36242694 PMC9886742

[4] Bawaskar HS Bawaskar PH. Oral diseases: a global public health challenge. Lancet. (2020) 395:185–6. doi: 10.1016/S0140-6736(19)33016-8 31954454

[5] IwasakiM MotokawaK WatanabeY ShirobeM InagakiH EdahiroA . Association between Oral frailty and nutritional status among community-dwelling older adults: the Takashimadaira study. J. Nutr. Health Aging. (2020) 24:1003–10. doi: 10.1007/s12603-020-1433-1, PMID: 33155629

[6] Minakuchi S Tsuga K Ikebe K Ueda T Tamura F Nagao K . Oral hypofunction in the older population: position paper of the Japanese Society of Gerodontology in 2016. Gerodontology. (2018) 35:317–24. doi: 10.1111/ger.12347, PMID: 29882364

[7] HoshinoD HiranoH EdahiroA MotokawaK ShirobeM WatanabeY . Association between Oral frailty and dietary variety among community-dwelling older persons: a cross-sectional study. J Nutr Health Aging. (2021) 25:361–8. doi: 10.1007/s12603-020-1538-633575729

[8] Watanabe Y Okada K Kondo M Matsushita T Nakazawa S Yamazaki Y. Oral health for achieving longevity. Geriatr Gerontol Int. (2020) 20:526–38. doi: 10.1111/ggi.13921 32307825

[9] Baba H Watanabe Y Miura K Ozaki K Matsushita T Kondoh M . Oral frailty and carriage of oral Candida in community-dwelling older adults (check-up to discover health with energy for senior residents in Iwamizawa; CHEER Iwamizawa). Gerodontology. (2022) 39:49–58. doi: 10.1111/ger.12621, PMID: 35098575

[10] Cruz-Moreira K Alvarez-Cordova L González-Palacios Torres C Chedraui P Jouvin J Jiménez-Moleón JJ . Prevalence of frailty and its association with oral hypofunction in older adults: a gender perspective. BMC Oral Health. (2023) 23:140. doi: 10.1186/s12903-023-02824-3, PMID: 36899360 PMC10007728

[11] Iwasaki M Motokawa K Watanabe Y Shirobe M Inagaki H Edahiro A . A two-year longitudinal study of the association between Oral frailty and deteriorating nutritional status among community-dwelling older adults. Int J Environ Res Public Health. (2020) 18:213. doi: 10.3390/ijerph18010213, PMID: 33396639 PMC7796237

[12] Iwasaki M Shirobe M Motokawa K Tanaka T Ikebe K Ueda T . Prevalence of Oral frailty and its association with dietary variety, social engagement, and physical frailty: results from the Oral frailty 5-item checklist. Geriatr Gerontol Int. (2024) 24:371–7. doi: 10.1111/ggi.14846 38390632

[13] Ohara Y Motokawa K Watanabe Y Shirobe M Inagaki H Motohashi Y . Association of eating alone with oral frailty among community-dwelling older adults in Japan. Arch Gerontol Geriatr. (2020) 87:104014. doi: 10.1016/j.archger.2020.104014, PMID: 32000053

[14] Rostom A Dubé C Cranney A Saloojee N Sy R Garritty C . Celiac disease. Evid Rep Technol Assess. (2014) 104:1–6. PMC4781297 15346868

[15] Jüni P Witschi A Bloch R Egger M. The hazards of scoring the quality of clinical trials for meta-analysis. JAMA. (1999) 282:1054–60. doi: 10.1001/jama.282.11.1054 10493204

[16] Higgins JP Thompson SG Deeks JJ Altman DGJB. Measuring inconsistency in meta-analyses. BMJ. (2003) 327:557–60. doi: 10.1136/bmj.327.7414.557, 12958120 PMC192859

[17] Ayoob A Janakiram C. Prevalence of physical and oral frailty in geriatric patients in Kerala, India. J Oral Biol Craniofac Res. (2024) 14:158–63. doi: 10.1016/j.jobcr.2024.01.011, PMID: 38347898 PMC10859288

[18] Chew J Chia JQ Kyaw KK Fu JK Ang J Lim YP . Association of Oral Health with frailty, malnutrition risk and functional decline in hospitalized older adults: a cross-sectional study. J Frailty Aging. (2023) 12:277–83. doi: 10.14283/jfa.2023.33, PMID: 38008977

[19] Hasegawa Y Sakuramoto-Sadakane A Nagai K Tamaoka J Oshitani M Ono T . Does oral hypofunction promote social withdrawal in the older adults? A longitudinal survey of elderly subjects in rural Japan. Int J Environ Res Public Health. (2020) 17:1–11. doi: 10.3390/ijerph17238904 PMC7731335 33266111

[20] Ishii M Yamaguchi Y Hamaya H Iwata Y Takada K Ogawa S . Influence of oral health on frailty in patients with type 2 diabetes aged 75 years or older. BMC Geriatr. (2022) 22:145. doi: 10.1186/s12877-022-02841-x 35183107 PMC8858474

[21] Iwasaki M Watanabe Y Motokawa K Shirobe M Inagaki H Motohashi Y . Oral frailty and gait performance in community-dwelling older adults: findings from the Takashimadaira study. J Prosthodont Res. (2021) 65:467–73. doi: 10.2186/jpr.JPR_D_20_00129, PMID: 33612666

[22] Kamdem B Seematter-Bagnoud L Botrugno F Santos-Eggimann B. Relationship between oral health and Fried's frailty criteria in community-dwelling older persons. BMC Geriatr. (2017) 17:1–8. doi: 10.1186/s12877-017-0568-3 28764647 PMC5539633

[23] Kamide N Ando M Murakami T Sawada T Hata W Sakamoto M. The association of oral frailty with fall risk in community-dwelling older adults: a cross-sectional, observational study. Eur Geriatric Med. (2024) 15:279–83. doi: 10.1007/s41999-023-00863-1, PMID: 37697213

[24] Kugimiya Y Watanabe Y Ueda T Motokawa K Shirobe M Igarashi K . Rate of oral frailty and oral hypofunction in rural community-dwelling older Japanese individuals. Gerodontology. (2020) 37:342–52. doi: 10.1111/ger.12468, PMID: 32141117

[25] Nagatani M Tanaka T Son BK Kawamura J Tagomori J Hirano H . Oral frailty as a risk factor for mild cognitive impairment in community-dwelling older adults: Kashiwa study. Exp Gerontol. (2023) 172:112075. doi: 10.1016/j.exger.2022.112075, PMID: 36581224

[26] Nishimoto M Tanaka T Hirano H Watanabe Y Ohara Y Shirobe M . Severe periodontitis increases the risk of Oral frailty: a six-year follow-up study from Kashiwa cohort study. Geriatrics. (2023) 8:25. doi: 10.3390/geriatrics8010025 36826367 PMC9956982

[27] Shimazaki Y Nonoyama T Tsushita K Arai H Matsushita K Uchibori N. Oral hypofunction and its association with frailty in community-dwelling older people. Geriatr Gerontol Int. (2020) 20:917–26. doi: 10.1111/ggi.14015, PMID: 32830417

[28] Tanaka T Hirano H Ikebe K Ueda T Iwasaki M Shirobe M . Oral frailty five-item checklist to predict adverse health outcomes in community-dwelling older adults: a Kashiwa cohort study. Geriatr Gerontol Int. (2023) 23:651–9. doi: 10.1111/ggi.14634 37661091 PMC11503571

[29] Velázquez-Olmedo LB Borges-Yáñez SA Andrade Palos P García-Peña C Gutiérrez-Robledo LM Sánchez-García S. Oral health condition and development of frailty over a 12-month period in community-dwelling older adults. BMC Oral Health. (2021) 21:1–10. doi: 10.1186/s12903-021-01718-6 34284766 PMC8290629

[30] Yoshida M Hiraoka A Takeda C Mori T Maruyama M Yoshikawa M . Oral hypofunction and its relation to frailty and sarcopenia in community-dwelling older people. Gerodontology. (2022) 39:26–32. doi: 10.1111/ger.12603, PMID: 34727388

[31] Diaz-Toro F Petermann-Rocha F Parra-Soto S Troncoso-Pantoja C Concha-Cisternas Y Lanuza F . Association between poor Oral health and frailty in middle-aged and older individuals: a cross-sectional National Study. J Nutr Health Aging. (2022) 26:987–93. doi: 10.1007/s12603-022-1858-9, PMID: 36437766

[32] Kimble R Papacosta AO Lennon LT Whincup PH Weyant RJ Mathers JC . The relationship of Oral health with progression of physical frailty among older adults: a longitudinal study composed of two cohorts of older adults from the United Kingdom and United States. J Am Med Dir Assoc. (2023) 24:468–8. doi: 10.1016/j.jamda.2022.11.022, PMID: 36584971 PMC10398566

[33] Komatsu R Nagai K Hasegawa Y Okuda K Okinaka Y Wada Y . Association between physical frailty subdomains and oral frailty in community-dwelling older adults. Int J Environ Res Public Health. (2021) 18:1–9. doi: 10.3390/ijerph18062931 PMC8001836 33809322

[34] Kuo YW Lee JD. Association between Oral frailty and physical frailty among rural middle-old community-dwelling people with cognitive decline in Taiwan: a cross-sectional study. Int J Environ Res Public Health. (2022) 19:2884. doi: 10.3390/ijerph19052884, PMID: 35270577 PMC8909940

[35] Shwe PS Thein PM Marwaha P Taege K Shankumar R Junckerstorff R. Anticholinergic burden and poor oral health are associated with frailty in geriatric patients undergoing inpatient rehabilitation: a cross-sectional study. Gerodontology. (2023) 40:213–9. doi: 10.1111/ger.12635, PMID: 35477932

[36] Tani A Mizutani S Oku S Yatsugi H Chu T Liu X . Association between oral function and physical pre-frailty in community-dwelling older people: a cross-sectional study. BMC Geriatr. (2022) 22:1–9. doi: 10.1186/s12877-022-03409-5 36056302 PMC9440534

[37] Watanabe Y Hirano H Arai H Morishita S Ohara Y Edahiro A . Relationship between frailty and Oral function in community-dwelling elderly adults. J Am Geriatr Soc. (2017) 65:66–76. doi: 10.1111/jgs.14355 27655106

[38] Cesari M Prince M Thiyagarajan JA De Carvalho IA Bernabei R Chan P . Frailty: an emerging public health priority. J Am Med Dir Assoc. (2016) 17:188–92. doi: 10.1016/j.jamda.2015.12.016 26805753

[39] Li T Shen Y Leng Y Zeng Y Li L Yang Z . The prevalence of oral frailty among older adults: a systematic review and meta-analysis. Eur Geriatr Med. (2024) 15:645–55. doi: 10.1007/s41999-023-00930-7, PMID: 38528284

[40] Hironaka S Kugimiya Y Watanabe Y Motokawa K Hirano H Kawai H . Association between oral, social, and physical frailty in community-dwelling older adults. Arch Gerontol Geriatr. (2020) 89:104105. doi: 10.1016/j.archger.2020.104105 32480111

[41] Tanaka T Hirano H Ohara Y Nishimoto M Iijima K. Oral frailty Index-8 in the risk assessment of new-onset oral frailty and functional disability among community-dwelling older adults. Arch Gerontol Geriatr. (2021) 94:104340. doi: 10.1016/j.archger.2021.104340, PMID: 33529863

[42] Castrejón-Pérez RC Jiménez-Corona A Bernabé E Villa-Romero AR Arrivé E Dartigues JF . Oral disease and 3-year incidence of frailty in Mexican older adults. J Gerontol A Biol Sci Med Sci. (2017) 72:951–7. doi: 10.1093/gerona/glw201, PMID: 28329793

[43] Soysal P Stubbs B Lucato P Luchini C Solmi M Peluso R . Inflammation and frailty in the elderly: a systematic review and meta-analysis. Ageing Res Rev. (2016) 31:1–8. doi: 10.1016/j.arr.2016.08.006, PMID: 27592340

[44] Ramsay SE Papachristou E Watt RG Tsakos G Lennon LT Papacosta AO . Influence of poor Oral health on physical frailty: a population-based cohort study of older British men. J Am Geriatr Soc. (2018) 66:473–9. doi: 10.1111/jgs.15175, PMID: 29266166 PMC5887899

[45] Zupo R Castellana F Bortone I Griseta C Sardone R Lampignano L . Nutritional domains in frailty tools: working towards an operational definition of nutritional frailty. Ageing Res Rev. (2020) 64:101148. doi: 10.1016/j.arr.2020.101148, PMID: 32827687

[46] Hussein S Kantawalla RF Dickie S Suarez-Durall P Enciso R Mulligan R. Association of Oral Health and Mini Nutritional Assessment in older adults: a systematic review with Meta-analyses. J Prosthodont Res. (2022) 66:208–20. doi: 10.2186/jpr.JPR_D_20_00207, PMID: 34261845

[47] Joshipura KJ Willett WC Douglass CW. The impact of edentulousness on food and nutrient intake. J Am Dent Assoc. (1996) 127:459–67. doi: 10.14219/jada.archive.1996.0237 8655866

[48] de Sire A Baricich A Ferrillo M Migliario M Cisari C Invernizzi M. Buccal hemineglect: is it useful to evaluate the differences between the two halves of the oral cavity for the multidisciplinary rehabilitative management of right brain stroke survivors? A cross-sectional study. Top Stroke Rehabil. (2020) 27:208–14. doi: 10.1080/10749357.2019.1673592, PMID: 31714187

[49] Lopez-Jornet P Saura-Perez M Llevat-Espinosa N. Effect of oral health dental state and risk of malnutrition in elderly people. Geriatr Gerontol Int. (2013) 13:43–9. doi: 10.1111/j.1447-0594.2012.00853.x, PMID: 22530802

[50] Hirata A Ishizaka M Sawaya Y Shiba T Urano T. Relationship between the swallowing function, nutritional status, and sarcopenia in elderly outpatients. Nihon Ronen Igakkai Zasshi. (2021) 58:134–42. doi: 10.3143/geriatrics.58.134 33627550

[51] Livingston G Sommerlad A Orgeta V Costafreda SG Huntley J Ames D . Dementia prevention, intervention, and care. Lancet. (2017) 390:2673–734. doi: 10.1016/S0140-6736(17)31363-6 28735855

[52] Tanaka T Takahashi K Hirano H Kikutani T Watanabe Y Ohara Y . Oral frailty as a risk factor for physical frailty and mortality in community-dwelling elderly. J Gerontol A Biol Sci Med Sci. (2018) 73:1661–7. doi: 10.1093/gerona/glx225 29161342

[53] Yoneyama F Okamoto T Tamura Y Ishii N Togashi K Soma O . Association between oral frailty and lower urinary tract symptoms among middle-aged and older adults in community-dwelling individuals: a cross-sectional study. Int Urol Nephrol. (2024) 56:1803–10. doi: 10.1007/s11255-023-03878-6, PMID: 38216828

