# Supplementary material for: Prevalence and unfavourable outcome of oral frailty in older adult: a systematic review and meta-analysis
Source: Front Public Health. 2024 Dec 18;12:1501793. doi: 10.3389/fpubh.2024.1501793 (PMC11688490; doi:10.3389/fpubh.2024.1501793)

**Table S1 Search strategy**

| **PubMed** |
| --- |
| #1 (oral frailty[Title/Abstract])) OR (oral frail[Title/Abstract])) OR (oral frail[Title/Abstract])) OR (oral health[MeSH])) |
| #2((“aged” [MeSH]) OR (elderly [Title/Abstract])) OR (older[Title/Abstract])) OR (ageing[Title/Abstract])) OR (old people [Title/Abstract])) OR ( older adult[Title/Abstract])) OR (elder[Title/Abstract])) |
| #3 #1 AND #2 |
| **Web of science** |
| #1 TS=( oral frailty OR oral frail OR oral frail* OR oral health) |
| #2 TS=(aged OR elderly OR older OR geriatric OR ageing OR old people OR older adult OR elder* |
| #3 #1 AND #2 |
| **Embase** |
| #1 'oral frailty' OR 'oral frail' OR 'oral frail*': ab, ti OR 'oral frail'/exp |
| #2 'aged' OR 'elderly' OR 'older' OR 'geriatric' OR 'ageing' OR 'old people' OR 'older adult' OR 'elder*': ab, ti OR 'aged'/exp |
| #3: #1 AND #2 |
| **Cochrane library** |
| #1 (oral frailty OR oral frail OR oral frail* OR oral health):ti,ab,kw |
| #2 (aged OR elderly OR older OR geriatric OR ageing OR old people OR older adult OR elder*):ti,ab,kw |
| #3 Search #1 AND #2 |
| **Scopus** |
| #1 TITLE-ABS ("oral frailty" OR "oral frail" OR "oral frail*") |
| #2 TITLE-ABS ("aged" OR "elderly" OR "older" OR "geriatric" OR "ageing" OR "old people" OR "older adult" OR "elder*") |
| #3 Search #1 AND #2 |
| **CINAHL** |
| #1 "oral frailty" OR "oral frail" OR "oral frail*" |
| #2 "aged" OR "elderly" OR "older" OR "geriatric" OR "ageing" OR "old people" OR "older adult" OR "elder*" |
| #3 Search #1 AND #2 |

**Table S2 Quality evaluation of cross-sectional studies**

| Study | 1.Define the source of information | 2.List inclusion and exclusion criteria | 3.Indicate time period used for identifying patients | 4.Indicate whether or subjects were consecutive if 0t population-based | 5.Indicate if evaluators of subjective components of study were masked to other aspects of the status of the participants | 6.Describe any assessments undertaken for quality assurance purposes | 7.Explain any patient exclusions from analysis | 8.Describe how confounding was assessed | 9.If applicable, explain how missing data were handled in the analysis | 10.Summarize patient response rates and completeness of data collection | 11.The percentage or number of patients for which incomplete data | Total |
| --- | --- | --- | --- | --- | --- | --- | --- | --- | --- | --- | --- | --- |
| Aneesa Ayoob, 2024 | 1 | 1 | 0 | 0 | 0 | 1 | 1 | 1 | 1 | 0 | 0 | 6 |
| Haruhisa Baba, 2022 | 1 | 1 | 1 | 1 | 0 | 1 | 1 | 1 | 1 | 1 | 0 | 9 |
| Chew, J, 2023 | 1 | 1 | 1 | 1 | 0 | 1 | 1 | 1 | 0 | 1 | 0 | 8 |
| Karla Cruz‑Moreira, 2023 | 1 | 1 | 1 | 1 | 0 | 1 | 1 | 1 | 1 | 1 | 0 | 9 |
| F. Diaz-Toro, 2022 | 1 | 1 | 1 | 1 | 0 | 1 | 0 | 1 | 1 | 0 | 0 | 7 |
| D. HOSHI0, 2021 | 1 | 1 | 1 | 1 | 0 | 1 | 1 | 1 | 1 | 1 | 0 | 9 |
| Masaki Ishii, 2022 | 1 | 1 | 1 | 1 | 0 | 1 | 1 | 1 | 1 | 1 | 0 | 9 |
| Masa0ri Iwasaki, 2020 | 1 | 1 | 1 | 1 | 0 | 1 | 1 | 1 | 1 | 1 | 0 | 9 |
| Masa0ri Iwasaki, 2021 | 1 | 1 | 1 | 1 | 0 | 1 | 1 | 1 | 1 | 1 | 0 | 9 |
| Masa0ri Iwasaki, 2024 | 1 | 1 | 1 | 1 | 0 | 1 | 1 | 1 | 1 | 1 | 0 | 9 |
| Benedikta Kamdem, 2017 | 1 | 1 | 1 | 1 | 0 | 1 | 1 | 1 | 1 | 1 | 0 | 9 |
| Naoto Kamide, 2023 | 1 | 1 | 1 | 1 | 0 | 1 | 0 | 1 | 1 | 0 | 0 | 7 |
| Ryo Komatsu, 2021 | 1 | 1 | 1 | 1 | 0 | 1 | 0 | 1 | 1 | 1 | 0 | 8 |
| Yoshihiro Kugimiya, 2020 | 1 | 1 | 1 | 1 | 0 | 1 | 1 | 1 | 1 | 1 | 0 | 9 |
| Ya-Wen Kuo, 2022 | 1 | 1 | 1 | 1 | 0 | 1 | 1 | 1 | 1 | 1 | 0 | 9 |
| Yuki Ohara, 2020 | 1 | 1 | 1 | 1 | 0 | 1 | 1 | 1 | 1 | 1 | 0 | 9 |
| Yoshihiro Shimazaki, 2020 | 1 | 1 | 1 | 1 | 0 | 1 | 1 | 1 | 1 | 1 | 0 | 9 |
| Phu S. Shwe, 2023 | 1 | 1 | 1 | 1 | 0 | 1 | 1 | 1 | 1 | 0 | 0 | 8 |
| Asuka Tani, 2021 | 1 | 1 | 1 | 1 | 0 | 1 | 1 | 1 | 1 | 0 | 0 | 8 |
| Yutaka Watanabe, 2016 | 1 | 1 | 1 | 1 | 0 | 1 | 1 | 1 | 1 | 0 | 0 | 8 |
| Mitsuyoshi Yoshida, 2021 | 1 | 1 | 1 | 1 | 0 | 1 | 1 | 1 | 1 | 0 | 0 | 8 |

**Table S3 Quality evaluation of cohort studies**

| study | Representativeness of the exposed cohort | Selection of the non- exposed cohort | Ascertainment of exposure | Demonstration that outcome of interest was no present as start of study | Comparability of cohort on the basis of the design or analysis | Assessment of outcome | Was follow-up long enough for outcomes to occur | Adequacy of follow up of cohorts | total quality score |
| --- | --- | --- | --- | --- | --- | --- | --- | --- | --- |
| Yoko Hasegawa, 2020 | 1 | 1 | 1 | 1 | 2 | 1 | 1 | 1 | 9 |
| Masanori Iwasaki, 2020 | 1 | 1 | 1 | 1 | 2 | 1 | 1 | 1 | 9 |
| Rachel Kimble, 2023 | 1 | 1 | 1 | 1 | 2 | 1 | 1 | 1 | 9 |
| Miyuki Nagatani, 2023 | 1 | 1 | 1 | 1 | 2 | 1 | 1 | 1 | 9 |
| Misa Nishimoto | 1 | 1 | 1 | 1 | 2 | 1 | 1 | 1 | 9 |
| Tomoki Tanaka, 2023 | 1 | 1 | 1 | 1 | 2 | 1 | 1 | 1 | 9 |
| Laura Bárbara Velázquez‑Olmedo, 2021 | 1 | 1 | 1 | 1 | 2 | 1 | 1 | 1 | 9 |

**
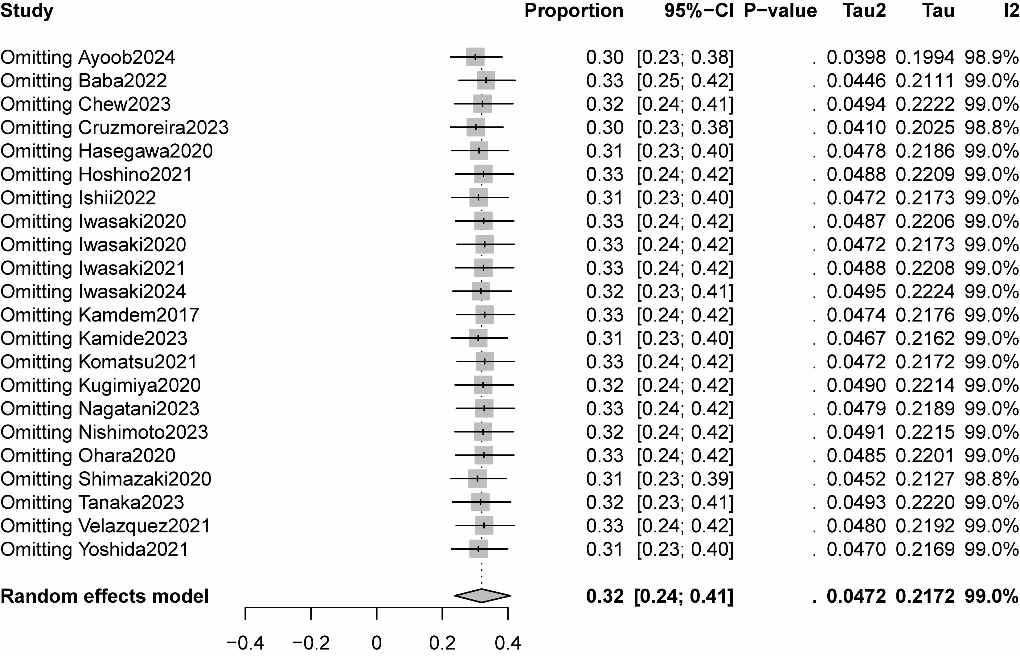
**

**Figure S1 Sensitivity analysis of for the prevalence of oral frailty**


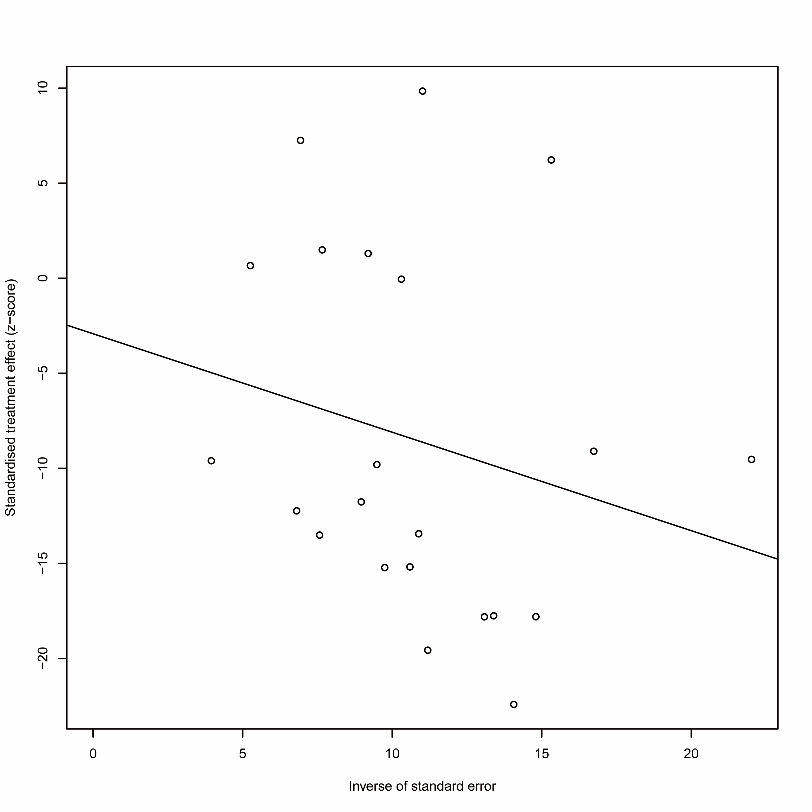


**Figure S2 The egger test of included studies**


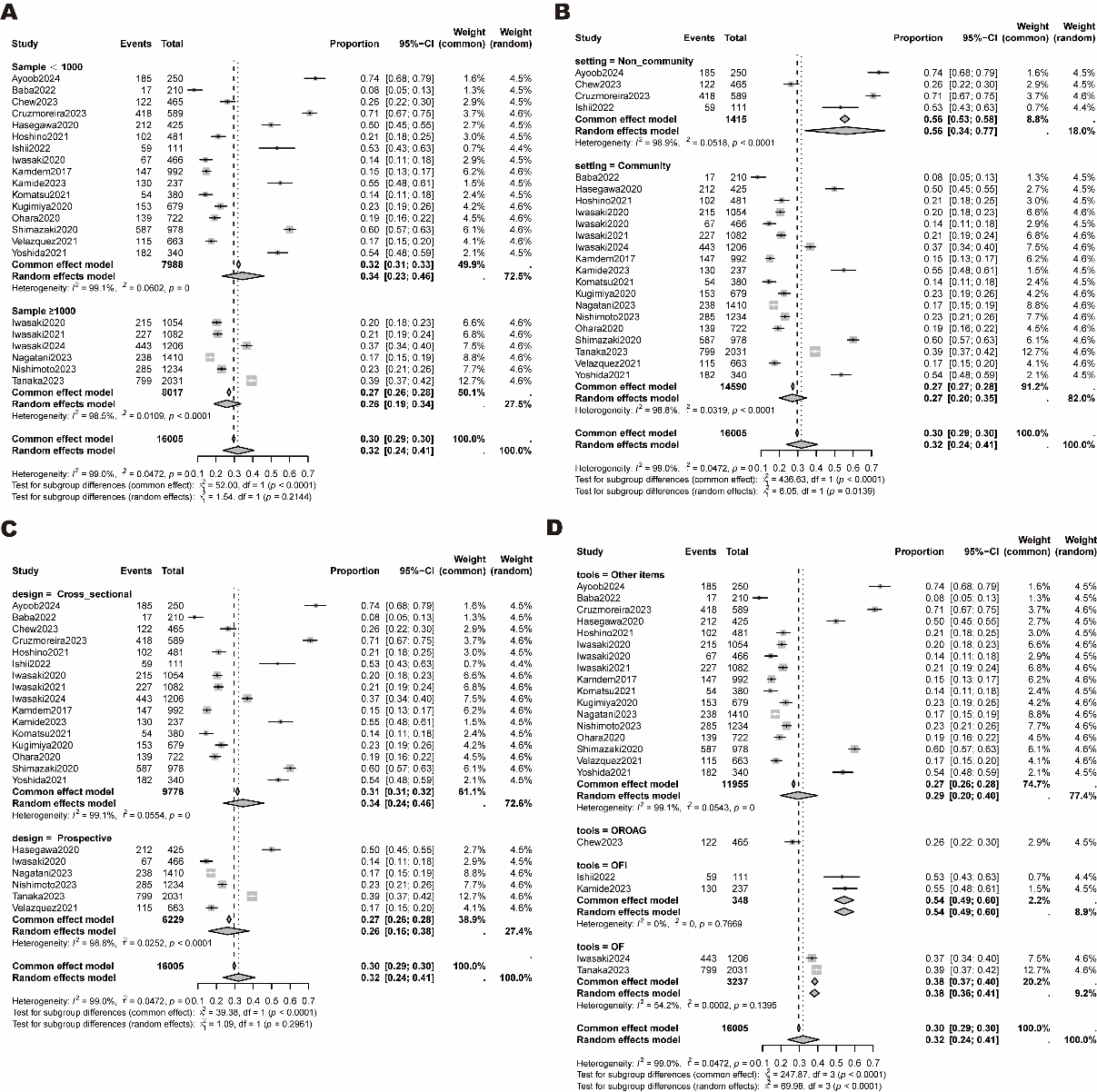


**Figure S3 Subgroup analyses for the prevalence of oral frailty**

**Figure S4 Funnel plot of the effect of oral frailty on physical frailty**


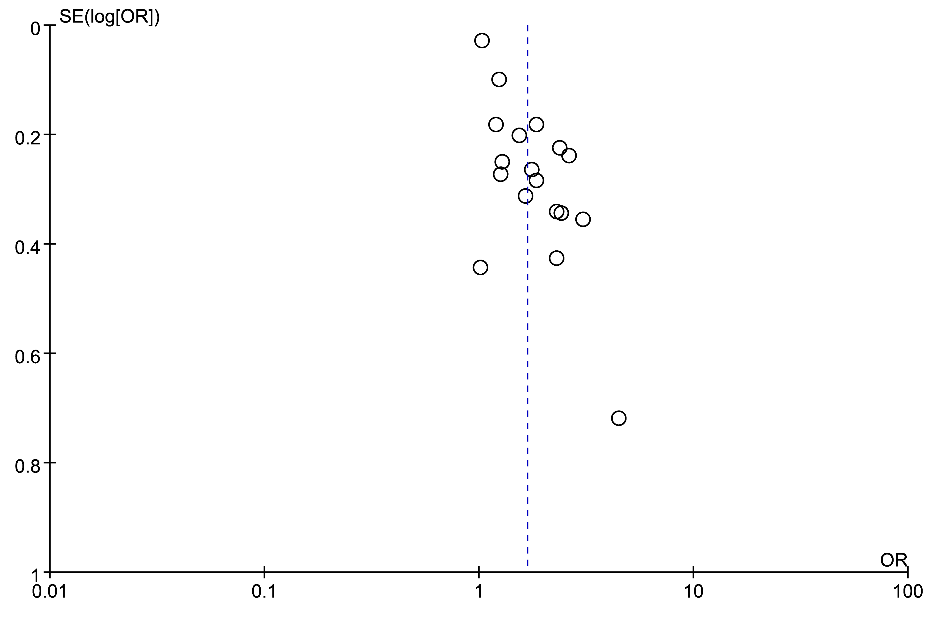

Supplement: Supplementary file 1 [file Table_1.docx]
